# ﻿Five new species of the trapdoor spider genus *Latouchia* Pocock, 1901 (Araneae, Halonoproctidae) from China

**DOI:** 10.3897/zookeys.1265.175629

**Published:** 2025-12-30

**Authors:** Yichao Xiong, Daiqin Li, Xin Xu

**Affiliations:** 1 College of Life Sciences, Hunan Normal University, Changsha, 410081, Hunan, China Hunan Normal University Changsha China; 2 Centre for Behavioural Ecology and Evolution, School of Life Sciences, Hubei University, 368 Youyi Road, Wuhan 430062, Hubei Province, China Hubei University Wuhan China

**Keywords:** COI, description, morphology, Mygalomorphae, taxonomy

## Abstract

Five new species of the trapdoor spider genus *Latouchia* Pocock, 1901 are described from southern China based on both morphological and molecular evidence: *L. jihe***sp. nov.** (♂♀), *L. wufeng***sp. nov.** (♂♀), *L. wuhan***sp. nov.** (♂♀), *L. yinggen***sp. nov.** (♂♀), and *L. zhangping***sp. nov.** (♂♀). The male and female of *L. jinyun* Hao, Yu & Zhang, 2025 are also redescribed from specimens collected in Nanchong City, Sichuan Province, China, located over 100 km from the type locality in Chongqing Municipality. Species delimitation is supported by genetic distance analyses of the mitochondrial DNA barcode gene (cytochrome c oxidase subunit I, COI), comparing the five new species with five previously described taxa. GenBank accession codes for the five new species and *L. jinyun* are provided to facilitate future identification and taxonomic research.

## ﻿Introduction

**Figure 1.**
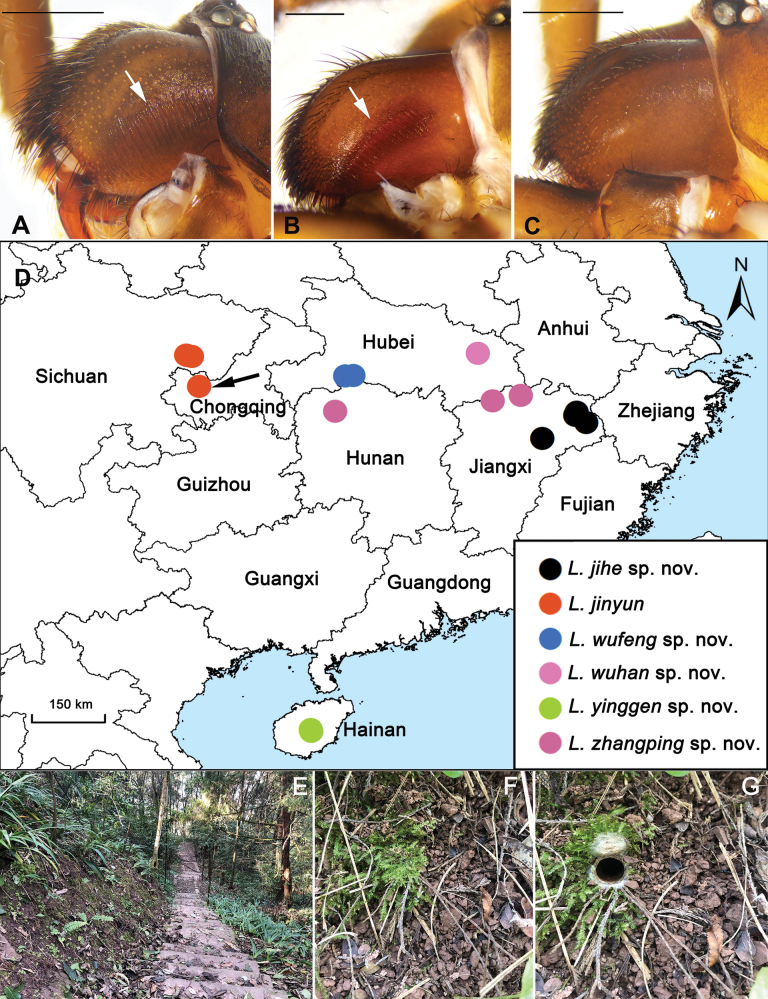
Stridulatory ridges on the retrolateral side of chelicerae, collection localities and microhabitat of *Latouchia* species in China. **A, B.***L. jihe* sp. nov., with white arrows indicating stridulatory ridges; **A.** Male; **B.** Female; **C.***L. zhangping* sp. nov., male chelicerae lacking stridulatory ridges; **D.** Map showing the collection localities of five new and one known *Latouchia* species in China; black arrow indicates the type locality of *L. jinyun* Hao, Yu & Zhang, 2025; **E.** Microhabitat; **F.** Burrow with trapdoor closed; **G.** Burrow with trapdoor open. Scale bars: 1 mm (**A–C**).

The trapdoor spider genus *Latouchia* Pocock, 1901 (Mygalomorphae, Halonoproctidae, Ummidiinae) (Godwin et al. 2018) currently includes 30 described species and one subspecies, all restricted to Asia, including 17 species and one subspecies recorded from China (WSC 2025). Of these, 19 species and one subspecies were known from both sexes, nine from females only, and two from males only (WSC 2025). The type species, *L. fossoria* Pocock, 1901, was recently re-established after nearly a century of taxonomic uncertainty (Decae and Caranhac 2020). Moreover, the presence or absence of crescent-shaped stridulatory ridges on the retrolateral surface of the chelicerae (Fig. 1A–C) has been proposed as an important character for distinguishing species groups—or potentially even separate genera—within *Latouchia* (Decae 2019).

Recent work by Hao and colleagues (2025) described five new *Latouchia* species from China and provided corresponding COI barcode sequences. By integrating morphological comparisons with molecular data, we identified five additional new species and confirmed the presence of the previously described species, *L. jinyun* Hao, Yu & Zhang, 2025 outside its type locality. The species was originally described from Jinyun Mountain National Nature Reserve, Chongqing Municipality, while we collected specimens more than 100 km away in Nanchong, Sichuan Province (Fig. 1D).

Here, we describe five new species of *Latouchia* and redescribe *L. jinyun* based on detailed examination of male palpal and female genital morphology, complemented by molecular data. We also provide cytochrome c oxidase subunit I (COI) sequences for all six species to support species delimitation and facilitate future identification. Additionally, we calculate genetic distances within and among the five new species and five previously described taxa to strengthen species delimitation and facilitate future taxonomic work.

## ﻿Materials and methods

All specimens were collected alive by excavating spiders from their underground burrows built on slopes within various habitats (Fig. 1E–G) across southern China (Fig. 1D). Subadult individuals were transported to the laboratory and reared to maturity. Four right legs from each adult specimen were removed, preserved in absolute ethanol, and stored at –80 °C for DNA extraction. The remaining body parts were preserved in 80% ethanol as vouchers for morphological examination. All voucher specimens are deposited at the Centre for Behavioural Ecology and Evolution (CBEE), School of Life Sciences, Hubei University, Wuhan, Hubei Province, China.

Morphological examinations and dissections were performed under an Olympus SZ51 stereomicroscope. Female genitalia were removed and digested in 10 mg/mL pancreatin solution (Biosharp Company, Hefei, Anhui, China) for at least 3 h at room temperature to clear soft tissues. Male palps and female genitalia were photographed using a digital CCD camera mounted on an Olympus BX53 compound microscope, with image stacks assembled using Helicon Focus v. 6.7.1. All measurements were taken in millimetres using a Leica M205C stereomicroscope equipped with Leica Application Suite v. 4. Palp and leg measurements are presented as: total length (femur, patella, tibia, metatarsus [absent in palps], tarsus).

The total genome DNA was extracted from leg muscle tissue using the Animal Genomic DNA Isolation Kit (Kangwei Biotech, China) following the manufacturer’s protocol. COI sequences were amplified using the primer pair LCO1490/HCO2198 (Folmer et al. 1994) following standard PCR conditions (Liu et al. 2019). COI sequences for five previously described species (*L. calcicola* Hao, Yu & Zhang, 2025, *L. jinyun*, *L. linmufu* Hao, Yu & Zhang, 2025, *L. wenchuan* Hao, Yu & Zhang, 2025, *L. yaoi* Hao, Yu & Zhang, 2025) were retrieved from the National Center for Biotechnology Information (NCBI). Sequence alignment was performed in Geneious Prime 2022 (https://www.geneious.com) with gap opening/extension penalties set to 24/3. Genetic distances were calculated in MEGA 11 (Tamura et al. 2021) using both Kimura 2-parameter (K2P) and *p*-distance nucleotide substitution models.

Abbreviations used in text are as follows:

**ALE** anterior lateral eye;

**AME** anterior median eye;

**AR** anterior eye row width;

**BL** body length (excluding chelicerae);

**CL** carapace length;

**CW** carapace width;

**EL** eye group length;

**LL** labium length;

**LW** labium width;

**MaxL** maxilla length;

**OL** opisthosoma length;

**OW** opisthosoma width;

**PLE** posterior lateral eye;

**PME** posterior median eye;

**PR** posterior eye row width;

**SL** sternum length;

**SW** sternum width.

## ﻿Taxonomy

### ﻿Genus Latouchia

Taxon classificationAnimaliaAraneaeHalonoproctidae

Pocock, 1901

E3C29BF7-9F8F-5750-832D-D362DBCE32E6

#### Type species.

*Latouchia fossoria* Pocock, 1901: 211, pl.21, fig. 2, female from China, by original designation; see Decae & Caranhac, 2020, 566, fig. 3, 17–26.

#### Diagnosis.

*Latouchia* can be distinguished from the other two genera of Ummidiinae, *Conothele* Thorell, 1878 and *Ummidia* Thorell, 1875, by the following combination of characters: (1) tibia III lacking a fully developed saddle depression; (2) female genitalia without a strongly sclerotized distal portion of the stalks (Figs 4I–K, 6G–J, 7G, 8G, 10, 12); and (3) male palp bearing a robust, stiff embolus (Figs 4A–C, 5C–H, 6A–E, 7A–F, 8A–F, 9, 11). For further details, see Decae et al. (2021).

### ﻿ Latouchia jihe sp. nov.

Taxon classificationAnimaliaAraneaeHalonoproctidae

E577558D-FD6C-53B6-A4B6-1DCB5EC575E0

https://zoobank.org/BF59C84A-F389-489F-B926-0A260BF9CE8B

**Figure 2.**
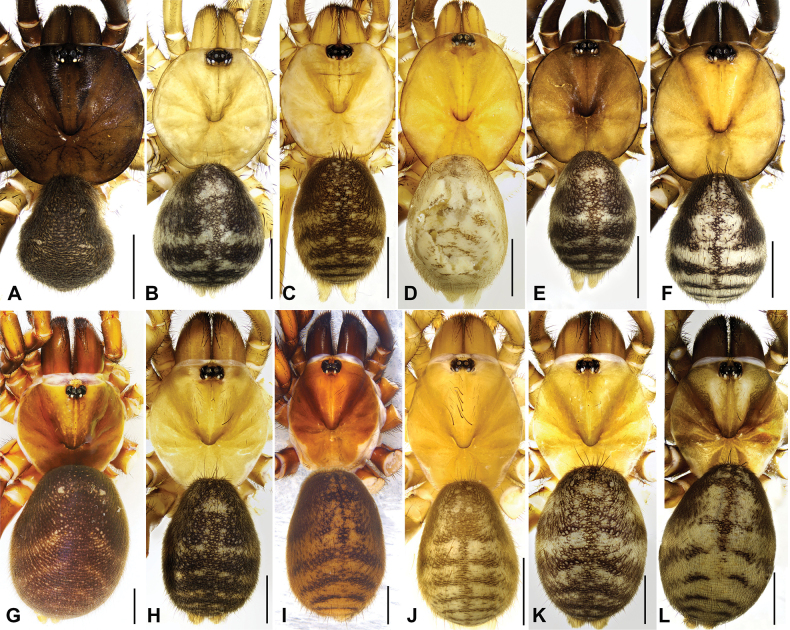
General somatic morphology of five new and one known *Latouchia* species. **A–F.** Male; **G–L.** Female; **A, G.***L. jihe* sp. nov. (HAL-2023-010, HAL-2023-032); **B, H.***L. wufeng* sp. nov. (HAL-2022-018A, HAL-2022-019B); **C, I.***L. wuhan* sp. nov. (XUC-2014-081, LH-2017-000); **D, J.***L. yinggen* sp. nov. (LH-2017-181, LH-2017-182); **E, K.***L. zhangping* sp. nov. (HAL-2023-098, HAL-2023-104); **F, L.***L. jinyun* (HAL-2023-139, HAL-2023-122). Scale bars: 2 mm (**A–L**).

**Figure 3.**
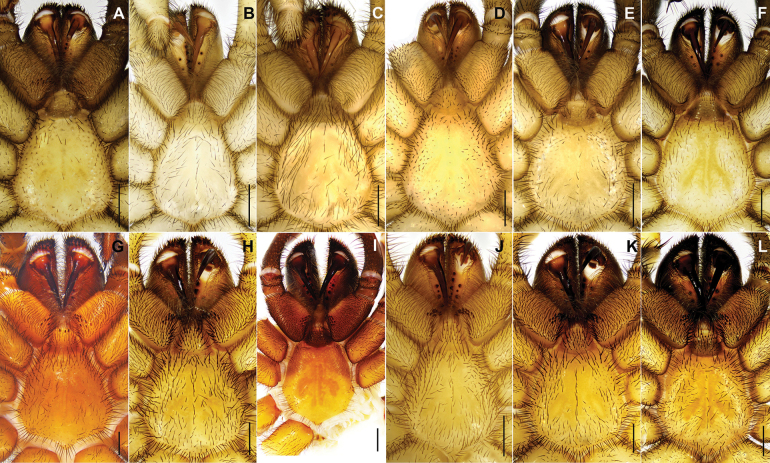
Chelicerae, labium, coxae of palp and sternum of five new and one known *Latouchia* species. **A–F.** Male; **G–L.** Female; **A, G.***L. jihe* sp. nov. (HAL-2023-010, HAL-2023-032); **B, H.***L. wufeng* sp. nov. (HAL-2022-018A, HAL-2022-019B); **C, I.***L. wuhan* sp. nov. (XUC-2014-081, LH-2017-000); **D, J.***L. yinggen* sp. nov. (LH-2017-181, LH-2017-182); **E, K.***L. zhangping* sp. nov. (HAL-2023-098, HAL-2023-104); **F, L.***L. jinyun* (HAL-2023-139, HAL-2023-122). Scale bars: 1 mm (**A–L**).

**Figure 4.**
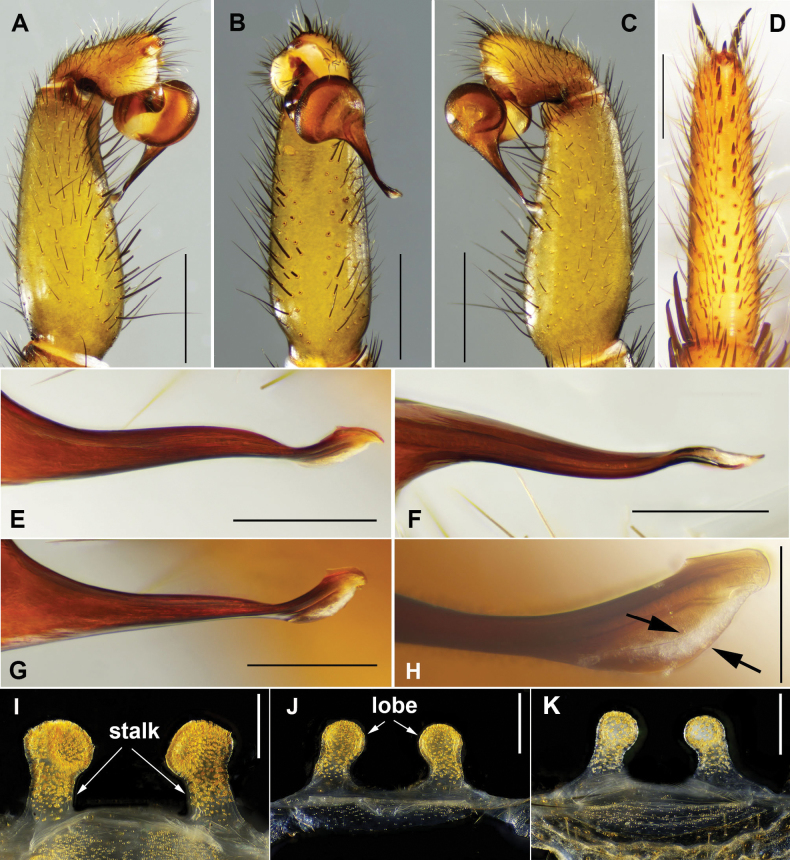
*Latouchia jihe* sp. nov., holotype male (**A–H**) and paratype females (**I–K**). **A–C, E–H.** Left male palp (HAL-2023-010): **A.** Prolateral view; **B.** Ventral view; **C.** Retrolateral view; **D.** Ventral side of tarsus I showing band of spinules; **E–H.** Embolus: **E, G, H.** Different angles of the embolus in retrolateral view; arrows indicate prolateral and retrolateral superior keels; **F.** Ventral view; **I–K.** Vulvae, dorsal view: **I.** HAL-2023-032; **J.** HAL-2023-036; **K.** HAL-2023-033. Scale bars: 1 mm (**A–C**); 0.5 mm (**D**); 0.3 mm (**E–G, I–K**); 0.15 mm (**H**).

Figs 1A, B, 2A, G, 3A, G, 4

#### Type material.

***Holotype*** • ♂ (HAL-2023-010, matured on 1 Dec. 2023), China, Jiangxi Province, Fuzhou City, Dongxiang District, Jihe Tower, 28 Aug. 2023, 28.20°N, 116.62°E, elev. 194 m, X. Xu, Y. Zhang, Y.X. Li, J.Y. Yuan leg. ***Paratype*** • 1♀ (HAL-2023-032), China, Jiangxi Province, Shangrao City, Guangxin District, Zhengfang Town, Lou Village, 30 Aug. 2023, 28.69°N, 117.90°E, elev. 135 m. • 1♀ (HAL-2023-033), China, Jiangxi Province, Shangrao City, Dexing City, Fenghuanghu Scenic Area, 31 Aug. 2023, 28.92°N, 117.60°E, elev. 71 m. • 1♀ (HAL-2023-036), China, Jiangxi Province, Shangrao City, Dexing City, Sizhou Town, Yejiazhuang Village, 31 Aug. 2023, 29.03°N, 117.63°E, elev. 118 m. X. Xu, Y. Zhang, Y.X. Li, J.Y. Yuan leg.

#### Diagnosis.

**Figure 5.**
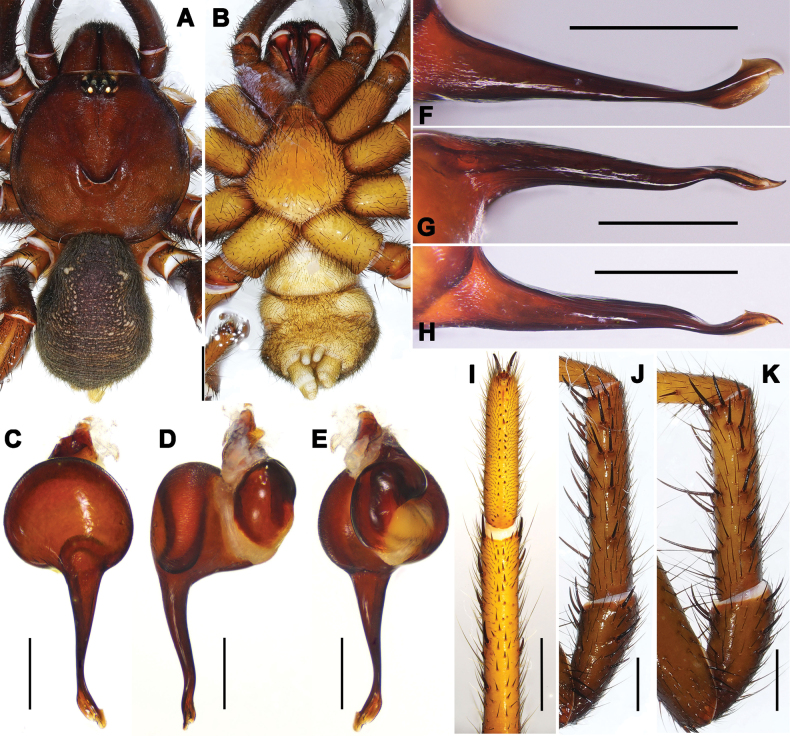
*Latouchia pavlovi* Schenkel, 1953, topotype male (KYUARA#2031, collected from Qingdao, Shandong). **A, B.** General somatic morphology; **A.** Dorsal view; **B.** Ventral view; **C–H.** Left male palp; **C.** Ventral view; **D.** Retrolateral view; **E.** Dorsal view; **F–H.** Embolus in different angles; **I.** Ventral side of tarsus I showing band of spinules; **J.** Patella and tibia of left leg I; **K.** Patella and tibia of left leg II. Scale bars: 2 mm (**A, B**); 0.5 mm (**C–H**); 1 mm (**I–K**).

This species can be distinguished from all other *Latouchia* species, except *L. davidi* (Simon, 1886), *L. pavlovi* Schenkel, 1953, and *L. stridulans* Decae, 2019, by presence of stridulatory ridges on the retrolateral surface of the chelicerae in both sexes (Fig. 1A, B). Male of *L. jihe* sp. nov. can be distinguished from *L. pavlovi* by embolus with slightly wider opening (Fig. 4E vs Fig. 5G) and embolic apex with slightly smaller triangular apophysis in retrolateral view (Fig. 4G vs Fig. 5F); from *L. stridulans* by embolus with larger opening (Fig. 4E–H vs figs 19–22 in Decae 2019). Females of *L. jihe* sp. nov. differ from *L. stridulans* and *L. pavlovi* by relatively slender stalks and knob-shaped spermathecae (Fig. 4I–K vs fig. 18 in Decae 2019 and fig. 1 in Song and Hu 1982), and from *L. davidi* by longer stalks (Fig. 4I–K vs fig. 11 in Decae and Caranhac 2020).

#### Description.

**Male** (holotype, Figs 1A, 2A, 3A). Carapace dark brown. Cephalic region smoothly elevated. Eight eyes arranged in two rows: anterior eye row procurved, posterior row straight. Chelicerae dark brown with a series of stridulatory ridges on the retrolateral surface (Fig. 1A), promargin with seven teeth, retromargin with six teeth. Fovea procurved and deep. Sternum light yellow with glabrous sigilla. Labium and maxillae light brown, labium with one cuspule, two maxillae together bearing 31 cuspules along proximal edge (Fig. 3A). Opisthosoma brownish-black.

Measurements: BL 8.93, CL 4.68, CW 4.49, OL 3.94, OW 3.01; Eye group, EL 0.49, AR 0.87, PR 0.89, AME-AME 0.11, AME 0.17, PME-PME 0.26, PME 0.12, ALE 0.21, PLE 0.21; MaxL 1.62, LL 0.68, LW 0.77; SL 2.49, SW 2.29; leg I 12.51 (3.96, 1.89, 2.93, 2.00, 1.73), leg II 11.10 (3.05, 1.78, 2.09, 2.39, 1.79), leg III 10.61 (3.06, 1.65, 1.92, 2.25, 1.73), leg IV 13.53 (4.20, 1.71, 2.77, 2.99, 1.86). Patellae and tibiae of legs I and II with long, straight, strong spines; spines slightly thicker on leg I (Fig. 13A, B).

Palp. Palpal bulb simple and elliptic in ventral view; embolus thick at base and gradually tapering toward the tip, apex twisted with narrow lanceolate opening; small, triangular apophysis at tip in retrolateral view; prolateral and retrolateral superior keels well developed (Fig. 4A–C, E–H).

**Female** (HAL-2023-032, Figs 1B, 2G, 3G). Carapace brown. Cephalic region smoothly elevated. Eight eyes arranged as in male. Chelicerae similar in colour to carapace, with a series of stridulatory ridges on retrolateral surface (Fig. 1B); promargin with seven teeth, retromargin with five teeth. Fovea procurved and deep. Sternum light yellow with distinct, glabrous sigilla. Labium and maxillae brown; labium with three cuspules; two maxillae together bearing 35 cuspules along proximal edge (Fig. 3G). Opisthosoma brown with pale, regular blotches.

Measurements: BL 18.24, CL 6.39, CW 5.99, OL 9.23, OW 6.73; Eye group, EL 0.67, AR 1.07, PR 1.08, AME-AME 0.12, AME 0.15, PME-PME 0.33, PME 0.15, ALE 0.34, PLE 0.30; MaxL 2.51, LL 1.15, LW 1.14; SL 3.90, SW 3.79; palp 9.66 (3.50, 1.91, 2.10, 2.15), leg I 11.17 (3.71, 2.40, 2.51, 1.44, 1.11), leg II 10.40 (3.48, 2.30, 2.05, 1.39, 1.18), leg III 10.23 (3.34, 2.28, 1.90, 1.35, 1.36), leg IV 14.76 (4.61, 2.69, 2.81, 2.57, 2.08).

Vulva. Paired spermathecae knob-shaped and slightly inward; entire lobes and distal ¾ of stalks densely covered with glandular pores; basal stalks gradually widened and pore-less (Fig. 4I–K).

#### Variation.

Females vary in body size and the number of cheliceral teeth. Measurements for females (*N* = 3): BL 10.13–18.24, CL 4.41–6.39, CW 3.89–5.99, OL 5.21–9.23, OW 3.72–6.73. Cheliceral teeth: 6–8 (promargin) and 5–7 (retromargin). Spermathecae with spherical (Fig. 4I, J) or ellipsoidal (Fig. 4K) lobes.

#### Genetic distance.

The mean and maximum intraspecific genetic distances for *L. jihe* sp. nov. are 3.46%/3.36% and 4.62%/4.44%, respectively (K2P/*p*-distance). The minimum interspecific genetic distance is 16.92%/15.11%, between *L. jihe* sp. nov. and *L. yinggen* sp. nov. (Table 1). The interspecific genetic distances between the holotype of new species and five known *Latouchia* species are shown in Table 2. Specimen information for genetic analysis based on COI is provided in Table 3.

**Table 1.** Summary of genetic distances (COI) for *Latouchia* species. *N*, the number of specimens used in the analyses.

Species	Mean intraspecific K2P/*p*-distances	Maximum intraspecific K2P/*p*-distances	Minimum interspecific K2P/*p*-distances	Closest species
*L. jihe* sp. nov. (*N* = 4)	3.46/3.36%	4.62/4.44%	16.92/15.11%	*L. yinggen* sp. nov.
*L. wufeng* sp. nov. (*N* = 6)	0.25/0.25%	0.75/0.74%	12.21/11.11%	*L. zhangping* sp. nov.
*L. wuhan* sp. nov. (*N* = 5)	0/0%	0/0%	13.80/12.58%	*L. zhangping* sp. nov.
*L. yinggen* sp. nov. (*N* = 4)	0/0%	0/0%	16.17/14.50%	*L. linmufu*
*L. zhangping* sp. nov. (*N* = 9)	1.52/1.49%	3.04/2.96%	12.21/11.11%	*L. wufeng* sp. nov.
*L. jinyun* (*N* = 29)	1.11/1.09%	2.42/2.37%	14.05/12.74%	*L. wuhan* sp. nov.

**Table 2.** Interspecific genetic distances among five new (holotype) and five known (holotype or paratype) *Latouchia* species based on COI sequences. The lower-left and upper-right matrices show genetic distance (%) calculated using the K2P and *p*-distance substitution models, respectively.

	Species	1	2	3	4	5	6	7	8	9	10
1	*L. jihe* sp. nov.		18.44	18.15	15.63	18.14	20.06	18.58	20.23	15.34	16.22
2	*L. wufeng* sp. nov.	21.31		15.73	19.03	11.21	19.47	15.78	21.18	18.58	18.73
3	*L. wuhan* sp. nov.	20.94	17.79		16.64	12.86	16.79	13.92	17.75	17.25	16.04
4	*L. yinggen* sp. nov.	17.59	22.20	18.94		17.26	18.73	18.14	14.50	15.93	16.96
5	*L. zhangping* sp. nov.	20.92	12.36	14.15	19.71		17.55	14.45	19.66	18.44	16.52
6	*L. calcicola*	23.48	22.75	19.12	21.64	20.12		19.03	19.47	20.21	18.88
7	*L. jinyun*	21.53	17.90	15.57	21.10	16.17	22.08		19.47	19.17	18.29
8	*L. linmufu*	23.66	25.21	20.32	16.17	22.95	22.68	22.84		20.23	21.37
9	*L. wenchuan*	17.20	21.56	19.69	18.02	21.37	23.71	22.35	23.71		12.68
10	*L. yaoi*	18.34	21.78	18.11	19.34	18.76	21.84	21.07	25.29	14.08	

**Table 3.** Specimens used for genetic distance analyses: specimen label, taxon name, collection locality with coordinates and GenBank accession numbers.

Specimen code	Sex	Species	Locality	Coordinates	Elevation (m)	COI GenBank accession
HAL-2023-010	Male	*L. jihe* sp. nov.	Fuzhou, Jiangxi	28.20, 116.62	194	PX504931
HAL-2023-032	Female	*L. jihe* sp. nov.	Shangrao, Jiangxi	28.69, 117.90	135	PX504965
HAL-2023-033	Female	*L. jihe* sp. nov.	Shangrao, Jiangxi	28.92, 117.60	71	PX504929
HAL-2023-036	Female	*L. jihe* sp. nov.	Shangrao, Jiangxi	29.03, 117.63	118	PX504930
HAL-2022-18A	Male	*L. wufeng* sp. nov.	Yichang, Hubei Pro	30.16, 110.77	1099	PX504970
HAL-2022-015	Female	*L. wufeng* sp. nov.	Yichang, Hubei Pro	30.17, 111.01	606	PX504968
HAL-2022-017	Female	*L. wufeng* sp. nov.	Yichang, Hubei Pro	30.16, 110.77	1099	PX504969
HAL-2022-019	Female	*L. wufeng* sp. nov.	Yichang, Hubei Pro	30.16, 110.77	1086	PX504971
HAL-2022-19B	Female	*L. wufeng* sp. nov.	Yichang, Hubei Pro	30.16, 110.77	1086	PX504972
HAL-2022-020	Female	*L. wufeng* sp. nov.	Yichang, Hubei	30.16, 110.77	1086	PX504973
XUC-2014-081	Male	*L. wuhan* sp. nov.	Wuhan, Hubei	30.85, 114.71	25	PX504974
XUC-2014-082	Female	*L. wuhan* sp. nov.	Wuhan, Hubei	30.85, 114.71	25	PX504975
XUC-2014-083	Juvenile	*L. wuhan* sp. nov.	Wuhan, Hubei	30.85, 114.71	25	PX504976
XUC-2014-084	Juvenile	*L. wuhan* sp. nov.	Wuhan, Hubei	30.85, 114.71	25	PX504977
LH-2017-000	Female	*L. wuhan* sp. nov.	Wuhan, Hubei	30.48, 114.35	33	PX626659
LH-2017-181	Male	*L. yinggen* sp. nov.	Qiongzhong, Hainan	19.03, 109.77	321	PX504932
LH-2017-181A	Juvenile	*L. yinggen* sp. nov.	Qiongzhong, Hainan	19.03, 109.77	321	PX504933
LH-2017-182	Female	*L. yinggen* sp. nov.	Qiongzhong, Hainan	19.08, 109.75	427	PX504934
LH-2017-183	Female	*L. yinggen* sp. nov.	Qiongzhong, Hainan	19.08, 109.75	427	PX504935
HAL-2023-098	Male	*L. zhangping* sp. nov.	Jiujiang, Jiangxi	29.38, 115.14	244	PX504979
HAL-2023-097	Female	*L. zhangping* sp. nov.	Jiujiang, Jiangxi	29.38, 115.14	244	PX504978
HAL-2023-100	Female	*L. zhangping* sp. nov.	Jiujiang, Jiangxi	29.38, 115.14	243	PX504980
HAL-2023-103	Female	*L. zhangping* sp. nov.	Jiujiang, Jiangxi	29.38, 115.14	243	PX504981
HAL-2023-104	Female	*L. zhangping* sp. nov.	Jiujiang, Jiangxi	29.38, 115.14	243	PX504982
HAL-2023-105	Female	*L. zhangping* sp. nov.	Jiujiang, Jiangxi	29.38, 115.14	247	PX504983
HAL-2019-111	Female	*L. zhangping* sp. nov.	Zhangjiajie, Hunan	29.05, 110.48	1416	PX504966
HAL-2019-111A	Female	*L. zhangping* sp. nov.	Zhangjiajie, Hunan	29.05, 110.48	1416	PX504967
LH-2017-030	Female	*L. zhangping* sp. nov.	Lushan, Jiangxi	29.55, 115.98	1134	PX504936
HAL-2023-139	Male	*L. jinyun*	Nanchong, Sichuan	30.80, 106.05	336	PX504956
HAL-2023-126	Male	*L. jinyun*	Nanchong, Sichuan	30.76, 106.21	561	PX504949
HAL-2023-108	Female	*L. jinyun*	Nanchong, Sichuan	30.79, 106.10	293	PX504937
HAL-2023-109	Female	*L. jinyun*	Nanchong, Sichuan	30.79, 106.10	293	PX504938
HAL-2023-110	Female	*L. jinyun*	Nanchong, Sichuan	30.79, 106.10	293	PX504939
HAL-2023-112	Female	*L. jinyun*	Nanchong, Sichuan	30.79, 106.10	293	PX504940
HAL-2023-113	Female	*L. jinyun*	Nanchong, Sichuan	30.79, 106.10	293	PX504941
HAL-2023-114	Female	*L. jinyun*	Nanchong, Sichuan	30.79, 106.10	293	PX504942
HAL-2023-115	Female	*L. jinyun*	Nanchong, Sichuan	30.79, 106.10	293	PX504943
HAL-2023-116	Female	*L. jinyun*	Nanchong, Sichuan	30.79, 106.10	293	PX504944
HAL-2023-122	Female	*L. jinyun*	Nanchong, Sichuan	30.76, 106.22	430	PX504945
HAL-2023-123	Female	*L. jinyun*	Nanchong, Sichuan	30.76, 106.21	561	PX504946
HAL-2023-124	Female	*L. jinyun*	Nanchong, Sichuan	30.76, 106.21	561	PX504947
HAL-2023-125	Female	*L. jinyun*	Nanchong, Sichuan	30.76, 106.21	561	PX504948
HAL-2023-127	Female	*L. jinyun*	Nanchong, Sichuan	30.76, 106.21	561	PX504950
HAL-2023-127A	Female	*L. jinyun*	Nanchong, Sichuan	30.76, 106.21	561	PX504951
HAL-2023-130	Female	*L. jinyun*	Nanchong, Sichuan	30.76, 106.21	561	PX504952
HAL-2023-133	Female	*L. jinyun*	Nanchong, Sichuan	30.76, 106.21	561	PX504953
HAL-2023-134	Female	*L. jinyun*	Nanchong, Sichuan	30.76, 106.21	538	PX504954
HAL-2023-137	Female	*L. jinyun*	Nanchong, Sichuan	30.80, 106.05	336	PX504955
HAL-2023-140	Female	*L. jinyun*	Nanchong, Sichuan	30.80, 106.05	336	PX504957
HAL-2023-141	Female	*L. jinyun*	Nanchong, Sichuan	30.80, 106.05	336	PX504958
HAL-2023-145	Female	*L. jinyun*	Nanchong, Sichuan	30.80, 106.05	336	PX504959
HAL-2023-146	Female	*L. jinyun*	Nanchong, Sichuan	30.80, 106.05	336	PX504960
HAL-2023-148	Female	*L. jinyun*	Nanchong, Sichuan	30.80, 106.05	336	PX504961
HAL-2023-149	Female	*L. jinyun*	Nanchong, Sichuan	30.80, 106.05	336	PX504962
HAL-2023-154	Female	*L. jinyun*	Nanchong, Sichuan	30.81, 106.04	333	PX504963
HAL-2023-155	Female	*L. jinyun*	Nanchong, Sichuan	30.81, 106.04	333	PX504964
KYUARA#1980	Female	*L. jinyun*	Jinyun Mountian, Chongqing	29.84, 106.40	376	*PQ585639
KYUARA#1978	Female	*L. calcicola*	Changjiang, Hainan	19.10, 109.02	122	*PQ585635
KYUARA#1981	Male	*L. linmufu*	Pingjiang, Hunan	28.96, 113.81	1500	*PQ585638
KYUARA#1983	Male	*L. wenchuan*	Wenchuan, Sichuan	31.47, 103.58	1377	*PQ585637
KYUARA#1984	Male	*L. yaoi*	Hanzhong, Shaanxi	33.25, 107.05	859	*PQ585636

* Sequences from GenBank.

#### Discussion.

Morphologically, *L. jihe* sp. nov. is most similar to *L. pavlovi*, which was originally described from Qingdao, Shandong Province on 6 July 1933 (Schenkel 1953) based solely on a male. The holotype of *L. pavlovi*, deposited at the “Museum Hoangho-Peiho” in Tientsin (currently corresponding to Beijiang Museum in Tianjin), is presumably lost. Based on information and photographs of a topotype male (Fig. 5) kindly provided by Kun Yu (Hebei University), *L. jihe* sp. nov. can be distinguished from *L. pavlovi* as detailed in the diagnosis.

#### Etymology.

The species epithet, a noun in apposition, refers to the type locality, Jihe Tower.

#### Distribution.

Jiangxi Province (Fuzhou, Shangrao).

### ﻿ Latouchia wufeng sp. nov.

Taxon classificationAnimaliaAraneaeHalonoproctidae

12F44718-10A7-5D1C-8D85-10B1CF450ADC

https://zoobank.org/399F138F-3670-4811-8F7F-97357E3BFAC7

**Figure 6.**
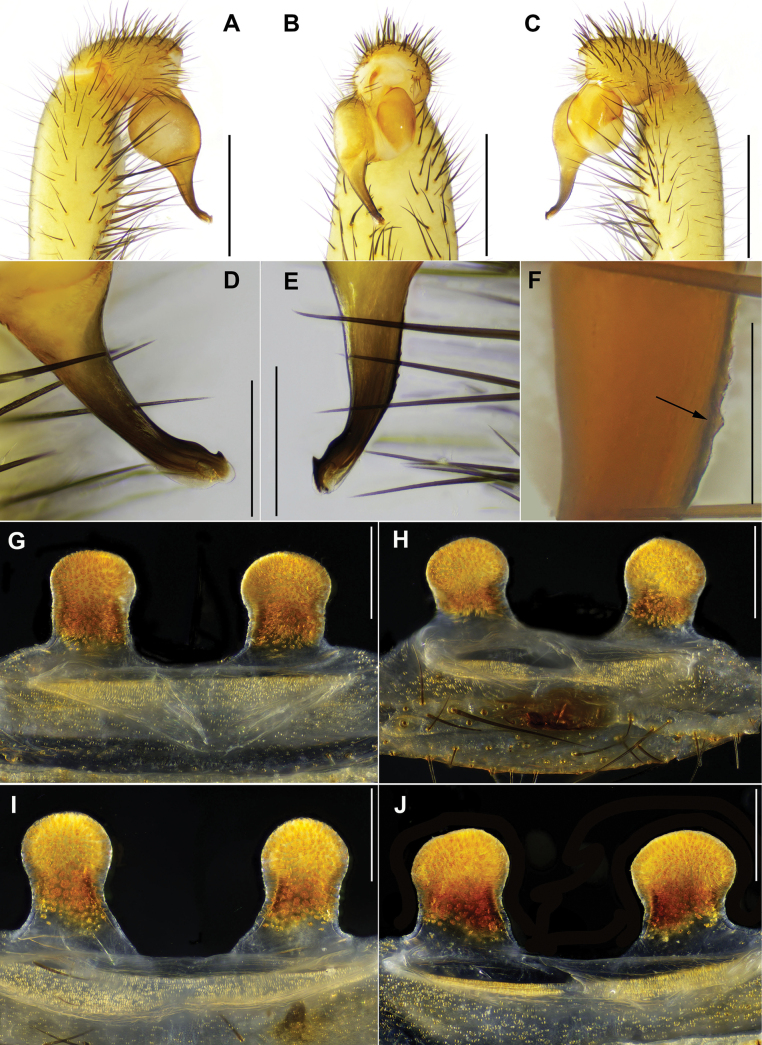
*Latouchia wufeng* sp. nov., holotype male (**A–F**) and paratype females (**G–J**). **A–F.** Left male palp (HAL-2022-018A): **A, D.** Prolateral view; **B.** Ventral view; **C, E, F.** retrolateral view; arrow indicates serrulate embolic keel; **G–J.** Vulvae, dorsal view: **G.** HAL-2022-019B; **H.** HAL-2022-019; **I.** HAL-2022-017; **J.** HAL-2022-020. Scale bars: 1 mm (**A–C**); 0.3 mm (**D, E, G–J**); 0.1 mm (**F**).

Figs 2B, H, 3B, H, 6

#### Type material.

***Holotype*** • ♂ (HAL-2022-018A, matured on 14 Aug. 2022), China, Hubei Province, Yichang City, Wufeng Tujia Autonomous County, Tangjiawan Village, 12 Aug. 2022, 30.16°N, 110.77°E, elev. 1099 m, X. Xu, Y. Zhan, Y. Zhang leg. ***Paratype*** • 6♀ (HAL-2022-017–021, 019B), same data as holotype. • 1♀ (HAL-2022-015) China, Hubei Province, Yichang City, Wufeng Tujia Autonomous County, Xiaowan Village, 12 Aug. 2022, 30.17°N, 111.01°E, elev. 606 m, X. Xu, Y. Zhan, Y. Zhang leg.

#### Diagnosis.

This species differs from *L. jihe* sp. nov., *L. davidi*, and *L. stridulans* by absence of stridulatory ridges on the chelicerae in both sexes. Male of *L. wufeng* sp. nov. resembles that of *L. zhangping* sp. nov. in having slightly thick embolus but can be distinguished by serrulate embolic keel visible in retrolateral view (Fig. 6F vs Fig. 9E) and by subterminal protuberance connected to a hook-shaped apex (Fig. 6D, E vs Fig. 9D, E). Females of *L. wufeng* sp. nov. differ from *L. zhangping* sp. nov. by spermathecae with less distinct constriction between lobes and stalks (Fig. 6G–J vs Fig. 10A–G).

#### Description.

**Male** (holotype, Figs 2B, 3B). Carapace yellowish brown. Cephalic region smoothly elevated. Eight eyes in two rows: anterior row procurved, posterior row straight. Chelicerae concolorous with carapace, promargin with six teeth, retromargin with three teeth. Fovea procurved and deep. Sternum pale yellow with sigilla. Labium and maxillae pale yellow, without cuspules (Fig. 3B). Opisthosoma brownish-black with regular light blotches.

Measurements: BL 9.48, CL 3.86, CW 4.07, OL 4.24, OW 3.83; Eye group, EL 0.45, AR 0.81, PR 0.87, AME-AME 0.12, AME 0.12, PME-PME 0.34, PME 0.09, ALE 0.29, PLE 0.23; MaxL 1.37, LL 0.61, LW 0.72; SL 2.29, SW 2.17; leg I 13.69 (4.39, 2.07, 3.03, 2.49, 1.71), leg II 12.07 (3.84, 1.94, 2.24, 2.27, 1.78), leg III 10.96 (3.01, 1.69, 1.79, 2.81, 1.66), leg IV 14.15 (3.99, 1.77, 2.95, 3.51, 1.93). Patellae and tibiae of legs I and II with stout spines, several bearing hooked tips (Fig. 13C, D).

Palp. Palpal bulb simple and pyriform in prolateral view; embolus thick, slightly curved at one-third of its length from apex; embolic keel serrulate in retrolateral view; apex with subterminal protuberance connected to a hook-shaped structure, both prolateral and retrolateral superior keels present (Fig. 6A–F).

**Female** (HAL-2022-019B, Figs 2H, 3H). Carapace yellowish brown, cephalic region elevated. Eyes in two rows: anterior row procurved, posterior row straight. Chelicerae brown, without stridulatory ridges, promargin with six teeth, retromargin with three teeth. Fovea procurved and deep. Sternum brown with indistinct sigilla. Labium and maxillae brown, labium with three cuspules, two maxillae together bearing 52 cuspules along proximal edge (Fig. 3H). Opisthosoma dark brown with light blotches.

Measurements: BL 12.80, CL 5.52, CW 4.96, OL 5.64, OW 4.12; Eye group, EL 0.57, AR 0.90, PR 0.98, AME-AME 0.14, AME 0.18, PME-PME 0.39, PME 0.11, ALE 0.30, PLE 0.25; MaxL 1.93, LL 0.89, LW 1.08; SL 3.07, SW 2.96; palp 8.65 (3.12, 1.69, 1.81, 2.03), leg I 11.37 (3.98, 2.29, 2.19, 1.57, 1.34), leg II 10.07 (3.13, 2.24, 1.72, 1.61, 1.37), leg III 9.45 (3.01, 2.26, 1.49, 1.57, 1.12), leg IV 13.21 (4.00, 2.27, 2.67, 2.48, 1.79).

Vulva. Spermathecae paired with indistinct subcentral constriction; entire spherical lobes and most of stalks densely covered with glandular pores (Fig. 6G–J).

#### Variation.

Females vary in body size and number of cheliceral teeth. Measurements for females (*N* = 7) are as follows: BL 12.23–16.55, CL 4.21–6.65, CW 4.14–6.15, OL 5.64–7.36, OW 4.12–5.16. Promarginal cheliceral teeth: 5–6. Spermathecae either parallel (Fig. 6G, J) or slightly tilted outward (Fig. 6H, I).

#### Genetic distance.

The intraspecific and interspecific genetic distances are provided in Tables 1, 2.

#### Etymology.

The species epithet, a noun in apposition, refers to the type locality.

#### Distribution.

Hubei Province (Yichang City).

### ﻿ Latouchia wuhan sp. nov.

Taxon classificationAnimaliaAraneaeHalonoproctidae

E69B9984-A2AA-55DE-A879-C37CFAC546C6

https://zoobank.org/009BD1AE-B3B7-462D-AB0C-166A8D940A50

**Figure 7.**
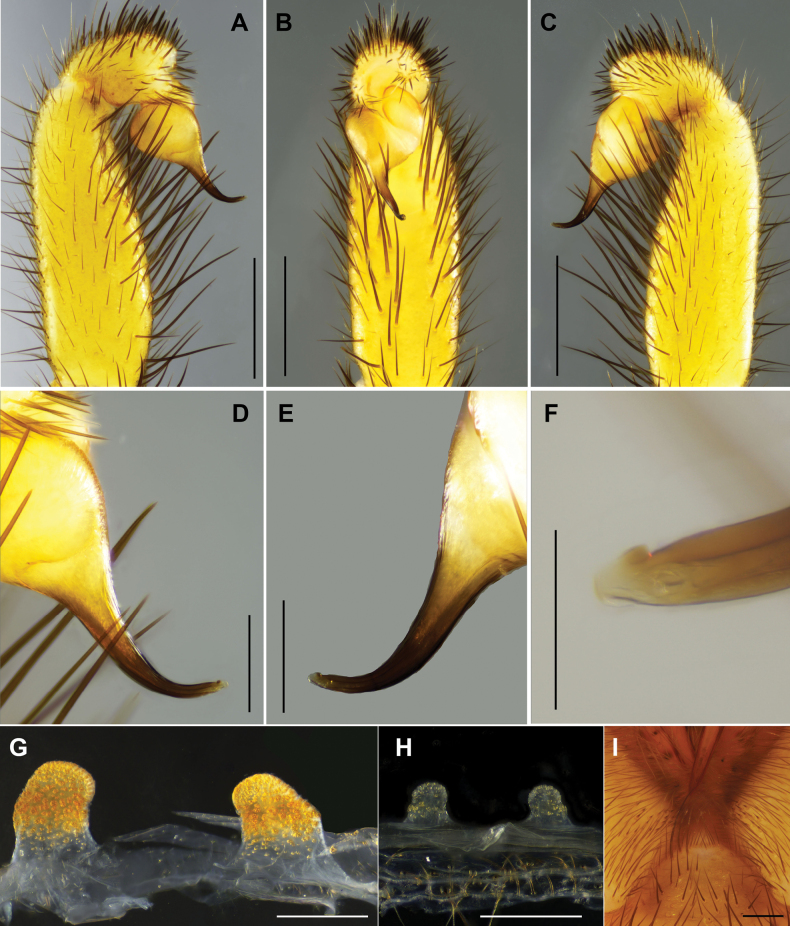
*Latouchia wuhan* sp. nov., holotype male (**A–F, I**) and paratype females (**G, H**). **A–F.** left male palp (XUC-2014-081): **A, D.** Prolateral view; **B.** Ventral view; **C, E, F.** Retrolateral view showing details of embolus; **G.** Vulva, dorsal view, LH-2017-000; **H.** Vulva of young female, dorsal view, XUC-2014-082; **I.** Thickened setae on maxillae. Scale bars: 1 mm (**A–C**); 0.3 mm (**D, E, G–I**); 0.1 mm (**F**).

Figs 2C, I, 3C, I, 7

#### Type material.

***Holotype*** • ♂ (XUC-2014-081), China, Hubei Province, Wuhan City, Hongshan District, Huazhong Agricultural University, 5 Sep. 2014, 30.85°N, 114.71°E, elev. 25 m, F.X. Liu, C. Xu leg. ***Paratype*** • 1♀ (LH-2017-000), same locality as holotype, 1 Mar. 2017, 30.48°N, 114.35°E, elev. 33 m, F.X. Liu, H. Liu leg. • 1 young female (XUC-2014-082), same data as holotype.

#### Diagnosis.

This species differs from *L. jihe* sp. nov., *L. davidi*, *L. pavlovi*, and *L. stridulans* by absence of stridulatory ridges on the chelicerae in both sexes. Male of *L. wuhan* sp. nov. resembles that of *L. jinyun* by distinctly curved embolus but can be distinguished from *L. jinyun* by shorter embolus (Fig. 7D–F vs Fig. 11D–I), and from *L. zhangping* sp. nov. by more slender embolus (Fig. 7D–F vs Fig. 9D, E). Female of *L. wuhan* sp. nov. can be distinguished from other *Latouchia* species by its incurved spermathecae, with lobes slightly narrower than stalks (Fig. 7G).

#### Description.

**Male** (holotype, Figs 2C, 3C, 7I). Carapace yellowish brown. Cephalic region elevated. Eyes in two rows: anterior row strongly procurved, posterior row straight. Chelicerae concolorous with carapace, without stridulatory ridges, promargin with five teeth, retromargin with four teeth. Fovea procurved and deep. Sternum yellow with glabrous sigilla. Labium and maxillae brown, two maxillae together bearing 40 short, basally thickened setae along proximal edge (Fig. 3C, 7I). Opisthosoma brown with regular light blotches.

Measurements: BL 10.52, CL 4.15, CW 4.12, OL 4.85, OW 3.50; Eye group, EL 0.50, AR 0.93, PR 0.95, AME-AME 0.15, AME 0.14, PME-PME 0.30, PME 0.13, ALE 0.27, PLE 0.22; MaxL 1.68, LL 1.05, LW 0.55; SL 2.53, SW 2.41; leg I 14.36 (4.49, 1.89, 3.39, 2.82, 1.77), leg II 13.02 (3.98, 2.32, 2.56, 2.41, 1.75), leg III 11.88 (3.12, 2.19, 1.81, 2.63, 2.13), leg IV 14.90 (4.42, 1.76, 3.30, 3.37, 2.05). Patellae and tibiae of legs I and II with stout spines; patellae II and tibiae II with more stout spines than patellae I and tibiae I, and most spines on tibiae II bearing hooked tips (Fig. 13E, F).

Palp. Palpal bulb simple and pyriform in prolateral view; embolus approximately equal in length to bulb, thick at base, tapering toward apex, and curved at one-third of its length from tip (Fig. 7A–F).

**Female** (LH-2017-000, Figs 2I, 3I). Carapace yellowish brown. Cephalic region elevated. Eyes in two rows: anterior row strongly procurved, posterior row procurved. Chelicerae brown, without stridulatory ridges, promargin with five teeth, retromargin with 10 teeth. Fovea procurved and deep. Sternum yellow with glabrous sigilla. Labium and maxillae yellowish brown, labium with one cuspule, two maxillae with 47 cuspules along proximal edge (Fig. 3I). Opisthosoma brown with regular light blotches.

Measurements: BL 18.40, CL 6.56 CW 5.16, OL 8.92, OW 5.71; Eye group, EL 0.90, AR 1.08, PR 0.99, AME-AME 0.13, AME 0.17, PME-PME 0.3, PME 0.15, ALE 0.43, PLE 0.32; MaxL 2.62, LL 0.96, LW 1.21; SL 3.39, SW 3.30; palp 10.10 (3.97, 1.69, 2.15, 2.29), leg I 11.56 (4.07, 2.21, 2.41, 1.71, 1.16), leg II 10.11 (3.56, 2.08, 1.78, 1.59, 1.10), leg III 8.76 (2.99, 1.69, 0.99, 1.68, 1.41), leg IV 14.62 (4.29, 2.84, 2.80, 2.85, 1.84).

Vulva. Spermathecae paired and incurved, with thick stalks; lobes slightly narrower than stalks, and both lobes and stalks covered with glandular pores (Fig. 7G, H).

#### Variation.

Females vary in body size. Measurements for females (*N* = 2): BL 9.49–18.40, CL 3.76–6.56, CW 3.22–5.16, OL 4.38–8.92, OW 3.07–5.71.

#### Genetic distance.

The intraspecific and interspecific genetic distances are provided in Tables 1, 2.

#### Etymology.

The species epithet, a noun in apposition, refers to the type locality.

#### Distribution.

Hubei Province (Wuhan City).

### ﻿ Latouchia yinggen sp. nov.

Taxon classificationAnimaliaAraneaeHalonoproctidae

0AAFB1C4-387C-5DA4-8B28-33CB3DC6F550

https://zoobank.org/A99B60CF-1AA8-4174-B8B7-6FF08F084501

**Figure 8.**
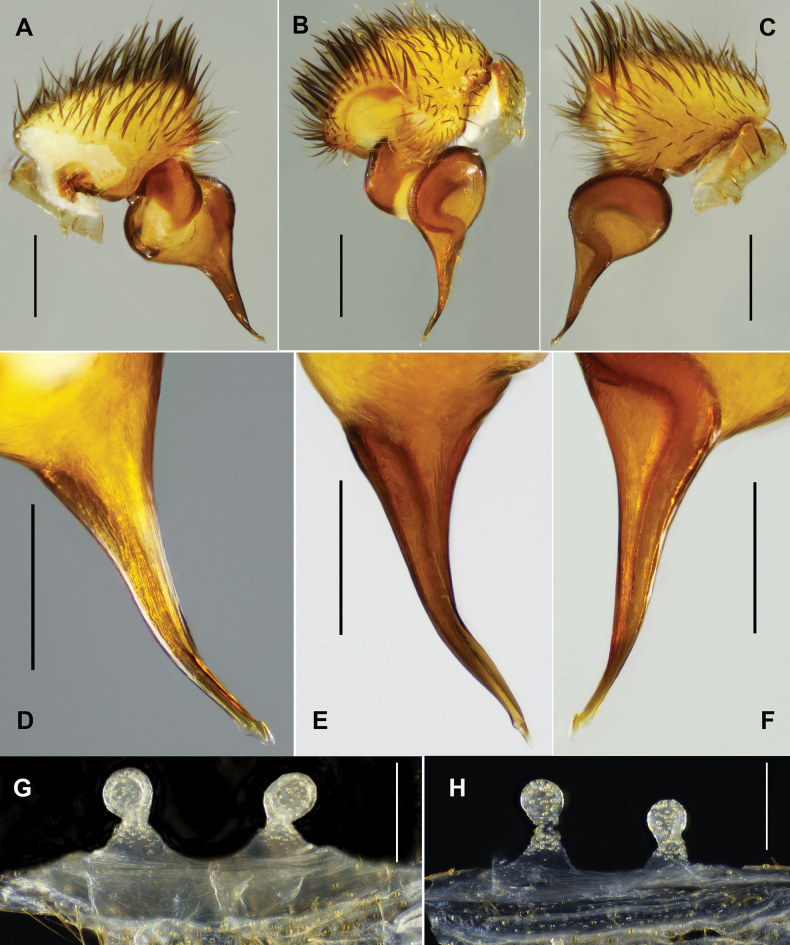
*Latouchia yinggen* sp. nov., holotype male (**A–F**) and paratype females (**G, H**). **A–F.** Right male palp (LH-2017-181): **A, D.** Prolateral view; **B, E.** Ventral view; **C, F.** Retrolateral view. **G, H.** Vulvae, dorsal view: **G.** LH-2017-182; **H.** LH-2017-183. Scale bars: 0.5 mm (**A–C**); 0.3 mm (**D–H**).

Figs 2D, J, 3D, J, 8

#### Type material.

***Holotype*** • ♂ (LH-2017-181), China, Hainan Province, Qiongzhong County, Yinggen Town, Nabai Village, 14 Aug. 2017, 19.03°N, 109.77°E, elev. 321 m, D. Li, F.X. Liu, X. Xu leg. ***Paratype*** • 2♀ (LH-2017-182, 183), China, Hainan Province, Qiongzhong County, Yinggen Town, Chaocan Village, 15 Aug. 2017, 19.09°N, 109.75°E, elev. 427 m, D. Li, F.X. Liu, X. Xu leg.

#### Diagnosis.

This species differs from *L. jihe* sp. nov., *L. davidi*, *L. pavlovi*, and *L. stridulans* by absence of stridulatory ridges on the chelicerae in both sexes. Male of *L. yinggen* sp. nov. can be distinguished from that of *L. linmufu* by embolus curved at one-third of its length from apex (Fig. 8D, E vs fig. 7G–J in Hao et al. 2025); from *L. calcicola* by lacking elevated embolic keel with slightly serrated edge in retrolateral view (Fig. 8C, F vs fig. 3A in Hao et al. 2025). Females of *L. yinggen* sp. nov. differ from those of *L. calcicola* and *L. linmufu* by parallel spermathecae with spherical lobes (Fig. 8G, H vs figs 2I, 7K in Hao et al. 2025). In addition, the new species differs from *L. calcicola* in both male and female by lacking band of spinules on ventro-posterior angle of coxae I–III (fig. 2C, D in Hao et al. 2025)

#### Description.

**Male** (holotype, Figs 2D, 3D). Carapace yellowish brown. Cephalic region smoothly elevated. Eight eyes in two rows: anterior row procurved, posterior row straight. Chelicerae black-brown, without stridulatory ridges, promargin with seven teeth, retromargin with five teeth. Fovea procurved and deep. Sternum yellowish brown with glabrous sigilla. Labium and maxillae yellowish brown, two maxillae together bearing approximately 37 cuspules along proximal edge (Fig. 3D). Opisthosoma pale yellow.

Measurements: BL 11.98, CL 5.27, CW 4.66, OL 5.30, OW 3.85; Eye group, EL 0.51, AR 0.84, PR 0.89, AME-AME 0.10, AME 0.18, PME-PME 0.32, PME 0.13, ALE 0.27, PLE 0.25; MaxL 1.80, LL 0.75, LW 0.95; SL 3.15, SW 2.79; leg I and II missing, leg III 14.91 (4.01, 2.22, 2.55, 3.97, 2.16), leg IV 21.16 (5.52, 2.48, 4.63, 5.80, 2.73).

Palp. Palpal bulb simple and elliptic in retrolateral view; embolus slightly thickened at base, tapering toward apex and curved at one-third of its length from tip; apex with triangular apophysis in retrolateral view, with both prolateral and retrolateral superior keels present (Fig. 8A–F).

**Female** (LH-2017-182, Figs 2J, 3J). Carapace yellowish brown. Cephalic region elevated. Eight eyes in two rows: anterior row procurved, posterior row straight. Chelicerae concolorous with carapace, without stridulatory ridges, promargin with six teeth, retromargin with five teeth. Fovea procurved and deep. Sternum yellow with indistinct sigilla. Labium and maxillae yellow, two maxillae with 31 cuspules along proximal edge (Fig. 3J). Opisthosoma yellowish brown with regular light blotches.

Measurements: BL 10.48, CL 4.65, CW 3.80, OL 4.84, OW 3.31; Eye group, EL 0.45, AR 0.79, PR 0.81, AME-AME 0.11, AME 0.13, PME-PME 0.26, PME 0.11, ALE 0.26, PLE 0.22; MaxL 1.64, LL 0.73, LW 0.87; SL 2.66, SW 2.37; palp 7.25 (2.51, 1.48, 1.59, 1.67), leg I 9.17 (2.95, 1.82, 1.98, 1.29, 1.13), leg II 8.24 (2.60, 1.63, 1.39, 1.41, 1.21), leg III 7.81 (2.35, 1.57, 1.09, 1.45, 1.35), leg IV 11.04 (3.19, 1.86, 2.17, 2.32, 1.50).

Vulva. Spermathecae paired and parallel, with distinct subcentral constriction; spherical lobes densely covered with glandular pores; stalks tapering in distal half (Fig. 8G, H).

#### Variation.

Females vary in body size. Measurements for females (*N* = 2): BL 10.48–11.64, CL 4.65–4.83, CW 3.71–3.80, OL 4.84–5.61, OW 3.31–4.12.

#### Genetic distance.

The intraspecific and interspecific genetic distances are provided in Tables 1, 2.

#### Etymology.

The species epithet, a noun in apposition, refers to the type locality.

#### Distribution.

Hainan Province (Qiongzhong).

### ﻿ Latouchia zhangping sp. nov.

Taxon classificationAnimaliaAraneaeHalonoproctidae

01C37D5C-3D8D-5B99-9F83-2F878377D3E6

https://zoobank.org/36EB6277-1ADE-403D-9041-1A0DE78ACEBD

**Figure 9.**
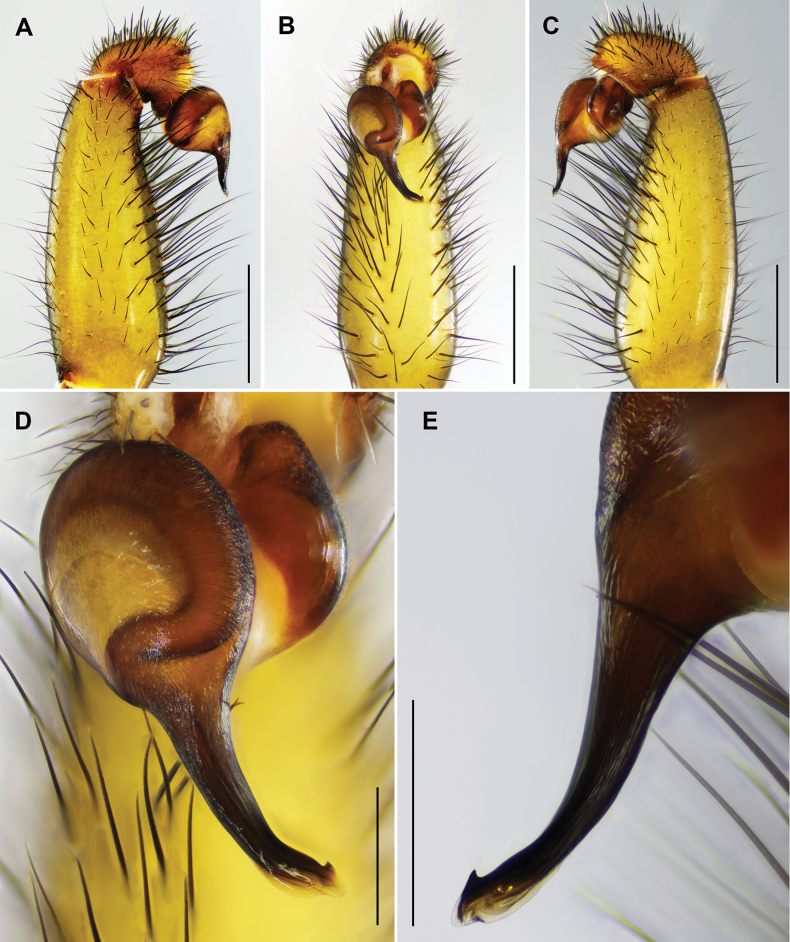
*Latouchia zhangping* sp. nov., holotype male, left male palp (HAL-2023-098): **A.** Prolateral view; **B, D.** Ventral view; **C, E.** Retrolateral view. Scale bars: 1 mm (**A–C**); 0.3 mm (**D–E**).

**Figure 10.**
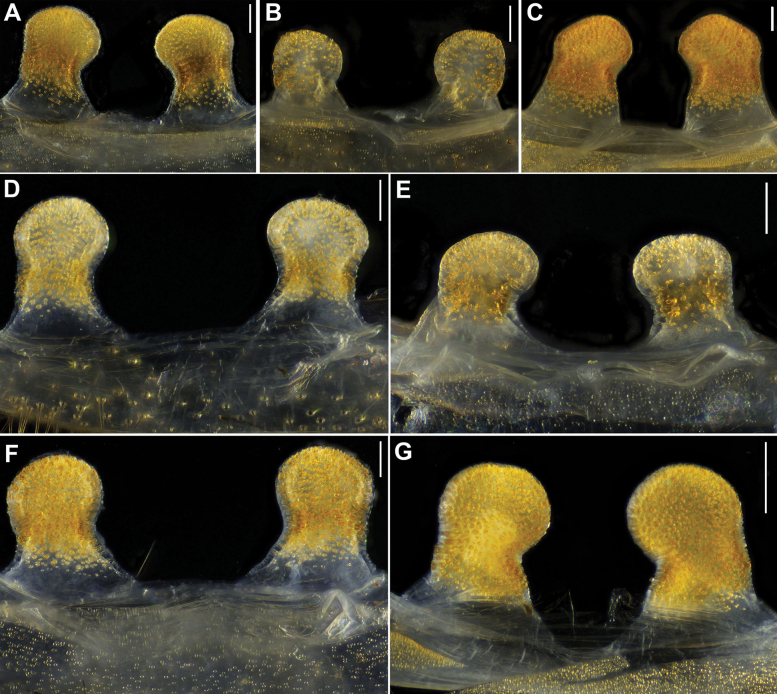
*Latouchia zhangping* sp. nov., paratypes (females), vulvae, dorsal view: **A.** HAL-2023-104; **B.** HAL-2019-111A; **C.** HAL-2019-111; **D.** HAL-2023-097; **E.** HAL-2023-100; **F.** HAL-2023-103; **G.** LH-2017-030. Scale bars: 0.1 mm (**A–G**).

Figs 1C, 2E, K, 3E, K, 9, 10

#### Type material.

***Holotype*** • ♂ (HAL-2023-098), China, Jiangxi Province, Jiujiang City, Wuning County, Henglu Town, Zhangping Village, 6 Sep. 2023, 29.38°N, 115.14°E, elev. 244 m, X. Xu, Y. Zhang, Y.X. Li, J.Y. Yuan leg. ***Paratype*** • 6♀ (HAL-2023-097, 099–104), same data as for the holotype. • 1♀ (LH-2017-030), China, Jiangxi Province, Jiujiang City, Lushan Scenic Area, 22 May 2017, 29.55°N, 115.98°E, elev. 1134 m, H. Liu, F.X. Liu leg. • 2♀ (HAL-2019-111, 111A), China, Hunan Province, Zhangjiajie City, Tianmenshan, 26 Sep. 2019, 29.05°N, 110.48°E, elev. 1416 m, X. Xu, D.Q. Li leg.

#### Diagnosis.

This species differs from *L. jihe* sp. nov., *L. davidi*, and *L. stridulans* by absence of stridulatory ridges on the chelicerae in both sexes. Male of *L. zhangping* sp. nov. can be distinguished from *L. wufeng* sp. nov. by embolus lacking serrulate embolic keel in retrolateral view, and by the lack of subterminal protuberance connected to a hook-shaped apex (Fig. 9D, E vs Fig. 6D–F). Females of *L. zhangping* sp. nov. differ from *L. wufeng* sp. nov. by inwardly knob-shaped spermathecae with distinct constriction between lobes and stalks (Fig. 10A–G vs Fig. 6G–J).

#### Description.

**Male** (holotype, Figs 1C, 2E, 3E). Carapace dark brown. Cephalic region smoothly elevated. Eight eyes in two rows: anterior row procurved, posterior row straight. Chelicerae dark brown without stridulatory ridges (Fig. 1C), promargin with five teeth, retromargin with three teeth. Fovea procurved and deep. Sternum yellow with glabrous sigilla. Labium and maxillae light brown, without cuspules (Fig. 3E). Opisthosoma dark brown with regular light blotches.

Measurements: BL 9.41, CL 4.46 CW 4.15, OL 4.45, OW 3.19; Eye group, EL 0.48, AR 0.78, PR 0.83, AME-AME 0.09, AME 0.17, PME-PME 0.30, PME 0.08, ALE 0.25, PLE 0.19; MaxL 1.45, LL 0.61, LW 0.71; SL 2.35, SW 2.36; leg I 14.39 (4.47, 2.15, 3.32, 2.80, 1.65), leg II 12.51 (4.05, 1.93, 2.61, 2.50, 1.42), leg III 10.96 (2.82, 1.83, 1.82, 2.76, 1.73), leg IV 14.58 (4.07, 1.82, 3.23, 3.49, 1.97). Patellae and tibiae of legs I and II with short and stout spines, most spines on tibiae I and II bearing slightly hooked tips (Fig. 13G, H).

Palp. Palpal bulb simple and elliptic in prolateral view; embolus thick, slightly curved at one-third of its length from apex, with a hook-shaped tip; both prolateral and retrolateral superior keels present (Fig. 9A–E).

**Female** (HAL-2023-104, Figs 2K, 3K). Carapace yellowish brown. Cephalic region smoothly elevated. Eight eyes in two rows: anterior row procurved, posterior row straight. Chelicerae brown without stridulatory ridges, promargin with five teeth, retromargin with five teeth. Fovea procurved and deep. Sternum brown with distinct glabrous sigilla. Labium and maxillae brown, and two maxillae together bearing 48 cuspules along proximal edge (Fig. 3K). Opisthosoma brown with regular light blotches.

Measurements: BL 14.84, CL 5.85 CW 5.33, OL 6.64, OW 5.14; Eye group, EL 0.63, AR 1.00, PR 1.05, AME-AME 0.13, AME 0.16, PME-PME 0.31, PME 0.14, ALE 0.31, PLE 0.27; MaxL 2.47, LL 1.21, LW 1.31; SL 3.41, SW 3.33; palp 10.19 (3.97, 1.98, 2.09, 2.15), leg I 11.83 (3.99, 2.42, 2.54, 1.73, 1.15), leg II 11.19 (3.66, 2.34, 2.35, 1.61, 1.23), leg III 10.93 (3.30, 2.32, 1.70, 1.91, 1.70), leg IV 14.37 (4.26, 2.67, 2.83, 2.74, 1.87).

Vulva. Spermathecae paired with knob-shaped apices, slightly inclined inward, with distinct constriction between lobes and stalks; spherical lobes densely covered with glandular pores extending over most of stalks (Fig. 10A–G).

#### Variation.

Females vary in body size and number of cheliceral teeth. Measurements for females (*N* = 9) are as follows: BL 9.70–21.70, CL 4.25–7.36, CW 3.71–7.47, OL 4.55–10.08, OW 3.49–8.75. The number of cheliceral teeth on retromargin ranges from 3–8. Female genitalia with longer (Fig. 10A, C, D, F, G) or shorter stalks (Fig. 10B, E).

#### Genetic distance.

The intraspecific and interspecific genetic distances are provided in Tables 1, 2.

#### Etymology.

The species epithet, a noun in apposition, refers to the type locality.

#### Distribution.

Jiangxi Province (Jiujiang City), Hunan Province (Zhangjiajie City).

### ﻿ Latouchia jinyun

Taxon classificationAnimaliaAraneaeHalonoproctidae

Hao, Yu & Zhang, 2025

55359F97-157A-5690-8D3C-6E8772683EDE

**Figure 11.**
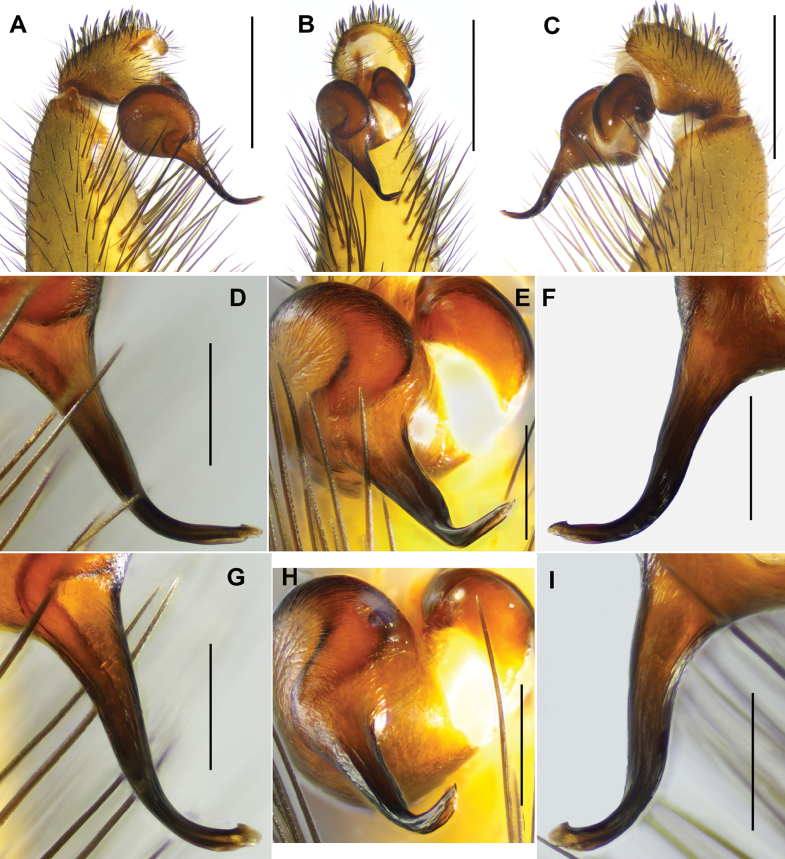
*Latouchia jinyun* Hao, Yu & Zhang, 2025, left male palp (A–F: HAL-2023-139, G–I: HAL-2023-126), **A, D, G.** prolateral view; **B, E, H.** ventral view; **C, F, I.** retrolateral view. Scale bars: 1 mm (**A–C**); 0.3 mm (**D–I**).

**Figure 12.**
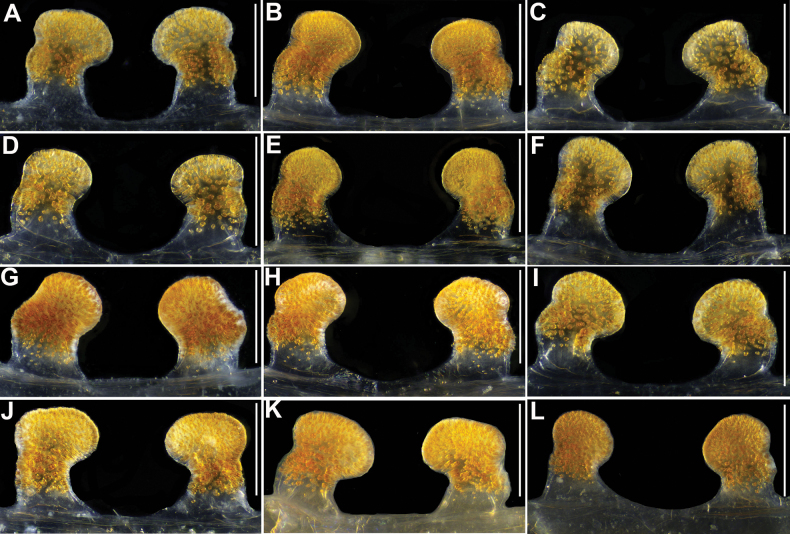
*Latouchia jinyun* Hao, Yu & Zhang, 2025, females, vulvae, dorsal view: **A.** HAL-2023-122; **B.** HAL-2023-112; **C.** HAL-2023-121; **D.** HAL-2023-108; **E.** HAL-2023-127; **F.** HAL-2023-127A; **G.** HAL-2023-137; **H.** HAL-2023-138; **I.** HAL-2023-140; **J.** HAL-2023-125; **K.** HAL-2023-146; **L.** HAL-2023-148. Scale bars: 0.3 mm (**A–L**).

Figs 2F, L, 3F, L, 11, 12

Latouchia jinyun Hao, Yu & Zhang, 2025: 19, figs 5A–I, 6A–F, 16d (described male and female, type locality: China, Chongqing Province, Beibei District, Jinyun Mountain National Nature Reserve, not examined).

#### Material examined.

• 10♀ (HAL-2023-107–110, 112–116, 118), China, Sichuan Province, Nanchong City, Gaoping District, Heming Mountain Scenic Area, 23 Dec. 2023, 30.79°N, 106.10°E, elev. 293 m, X. Xu leg. • 2♂14♀ (HAL-2023-121–127A, 128, 130, 131, 133–136, 136A), China, Sichuan Province, Nanchong City, Gaoping District, Laojun Town, Lingyun Mountain Scenic Area, 24 Dec. 2023, 30.76°N, 106.21°E, elev. 561 m. X. Xu, T.B. Yang leg. • 1♂14♀ (HAL-2023-137–141, 144–149, 151, 152, 154, 155), China, Sichuan Province, Nanchong City, Shunqing District, Xishan Scenic Area, 24 Dec. 2023, 30.80°N, 106.05°E, elev. 336 m, X. Xu, T.B. Yang leg.

#### Diagnosis.

*Latouchia jinyun* differs from *L. jihe* sp. nov., *L. davidi*, *L. pavlovi*, and *L. stridulans* by absence of stridulatory ridges on the chelicerae in both sexes. Males of *L. jinyun* can be distinguished from *L. wuhan* sp. nov. by longer embolus (Fig. 11A–I vs Fig. 7A–F). Females of *L. jinyun* differ from other *Latouchia* species by longer, inwardly curved stalks with distinct constriction between lobes and stalks (Fig. 12A–L).

#### Redescription.

**Male** (HAL-2023-139, Figs 2F, 3F). Carapace yellowish brown. Cephalic region smoothly elevated. Eight eyes in two rows: anterior row procurved, posterior row straight. Chelicerae dark brown without stridulatory ridges, promargin with five teeth, retromargin with three teeth. Fovea procurved and deep. Sternum yellow with obvious glabrous sigilla. Labium and maxillae brown, without cuspule (Fig. 3F). Opisthosoma brownish-black with regular light blotches.

**Figure 13.**
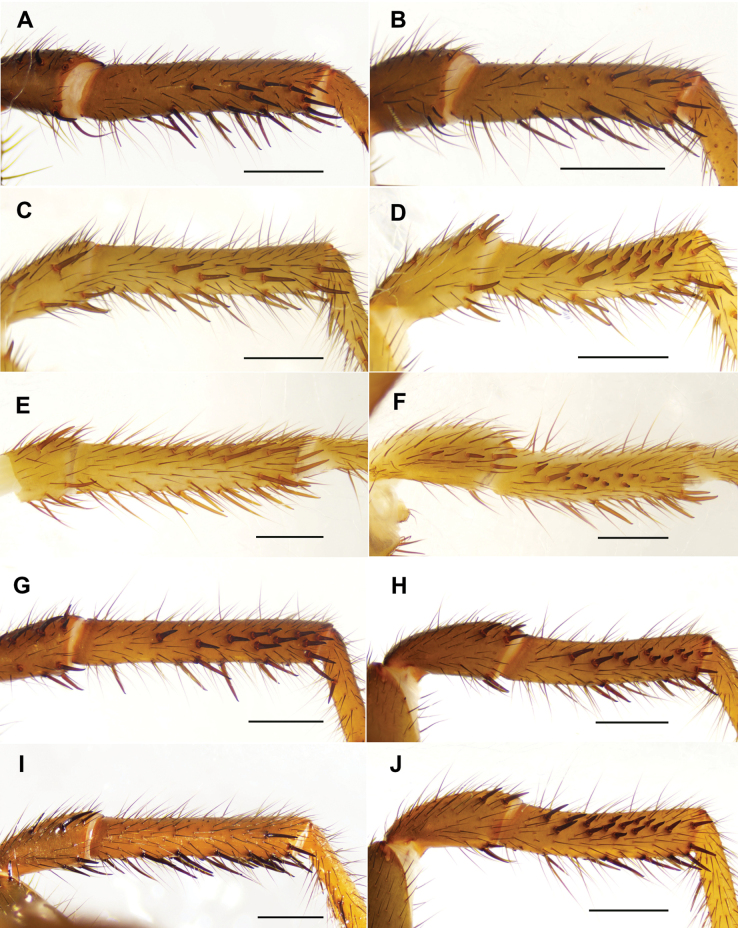
Spines on left patella and tibia I (**A, C, E, G, I**) and II (**B, D, F, H, J**) of males of different species. Prolateral view: **A, B.***Latouchia jihe* sp. nov.; **C, D.***Latouchia wufeng* sp. nov.; **E, F.***Latouchia wuhan* sp. nov.; **G, H.***Latouchia zhangping* sp. nov.; **I, J.***Latouchia jinyun* (HAL-2023-139. Scale bars: 1 mm (**A–J**).

Measurements: BL 9.64, CL 4.62 CW 4.23, OL 4.18, OW 3.21; Eye group, EL 0.51, AR 0.82, PR 0.98, AME-AME 0.11, AME 0.14, PME-PME 0.33, PME 0.11, ALE 0.30, PLE 0.28; MaxL 1.65, LL 0.56, LW 0.74; SL 2.80, SW 2.58; leg I 14.42 (4.46, 2.32, 3.13, 2.88, 1.63), leg II 13.09 (4.01, 2.27, 2.50, 2.67, 1.64), leg III 11.05 (2.97, 1.87, 1.49, 2.83, 1.89), leg IV 15.61 (4.31, 2.07, 3.23, 3.77, 2.23). Patellae and tibiae of legs I and II with stout spines; spines on tibiae II slightly thicker than those on tibiae I, most spines on tibiae II bearing hooked tips (Fig. 13I, J).

Palp. Palpal bulb simple and pyriform in prolateral view; embolus thick at the base, tapering to slender apex and curved at one-third of its length from hook-shaped tip; both prolateral and retrolateral superior keels present (Fig. 11A–I).

**Female** (HAL-2023-122, Figs 2L, 3L). Carapace yellowish brown. Cephalic region elevated. Eyes in two rows: anterior row procurved, posterior row straight. Chelicerae dark brown without stridulatory ridges, promargin with five teeth, retromargin with three teeth. Fovea procurved and deep. Sternum brown with obvious glabrous sigilla. Labium and maxillae brown, labium with one cuspule, two maxillae together with 61 cuspules along proximal edge (Fig. 3L). Opisthosoma brownish-black with regular light blotches.

Measurements: BL 13.44, CL 5.39 CW 4.67, OL 6.69, OW 4.63; Eye group, EL 0.79, AR 0.91, PR 0.96, AME-AME 0.13, AME 0.14, PME-PME 0.34, PME 0.12, ALE 0.32, PLE 0.30; MaxL 2.41, LL 0.84, LW 1.06; SL 3.35, SW 3.18; palp 8.37 (3.01, 1.44, 1.87, 2.05), leg I 11.12 (3.81, 2.31, 2.46, 1.41, 1.13), leg II 10.02 (3.34, 2.05, 1.93, 1.44, 1.26), leg III 8.85 (2.93, 1.95, 1.14, 1.46, 1.37), leg IV 13.40 (3.97, 2.34, 2.64, 2.64, 1.81).

Vulva. Paired spermathecae inclined inward, with distinct constriction between lobes and inwardly curved stalks; surfaces densely covered with glandular pores on both elliptical lobes and most of stalks (Fig. 12A–L).

#### Variation.

Males and females vary in body size and the number of cheliceral teeth. Measurements for males (*N* = 3): BL 9.64–10.48, CL 3.87–4.78, CW 3.75–4.36, OL 4.13–4.85, OW 2.74–3.31; Measurements for females (*N* = 38): BL 9.44–18.10, CL 3.59–6.75, CW 3.27–6.08, OL 4.33–8.79, OW 3.12–6.26. The number of cheliceral teeth on promargin ranges from 5–8.

#### Genetic distance.

The specimens from Nanchong, Sichuan are identified as *L. jinyun* because their genital morphology is identical to that of the type specimens from Jinyun Mountain, Chongqing. Genetic divergence between specimens from the two localities is also insufficient to justify treating Nanchong specimens as a separate species. The minimum and maximum genetic distances between the Nanchong specimens and the type specimen from Jinyun Mountain (GenBank accession code: PQ585635) are 1.65–2.42% (K2P) and 1.63–2.37% (*p*-distance), respectively. Within the Nanchong specimens, the minimum and maximum genetic distances range from 0% to 2.42% (K2P) and 0% to 2.37% (*p*-distance) (Table 1).

#### Distribution.

Chongqing Municipality, Sichuan Province (Nanchong City).

## Supplementary Material

XML Treatment for Latouchia

XML Treatment for Latouchia jihe

XML Treatment for Latouchia wufeng

XML Treatment for Latouchia wuhan

XML Treatment for Latouchia yinggen

XML Treatment for Latouchia zhangping

XML Treatment for Latouchia jinyun

## ﻿Additional information

### Use of AI

No use of AI was reported.

### Author ORCIDs

Yichao Xiong https://orcid.org/0009-0006-5709-8514

Daiqin Li https://orcid.org/0000-0001-8269-7734

Xin Xu https://orcid.org/0000-0001-5632-6622

### Data availability

All of the data that support the findings of this study are available in the main text.
